# Antibacterial Silver Nanomaterial Synthesis From *Mesoflavibacter zeaxanthinifaciens* and Targeting Biofilm Formation

**DOI:** 10.3389/fphar.2019.00801

**Published:** 2019-08-02

**Authors:** Mohammad Oves, Mohd Ahmar Rauf, Afzal Hussain, Huda A. Qari, Aftab Aslam Parwaz Khan, Pir Muhammad, Md Tabish Rehman, Mohammad Fahad Alajmi, Iqbal I. M. Ismail

**Affiliations:** ^1^Center of Excellence in Environmental Studies, King Abdulaziz University, Jeddah, Saudi Arabia; ^2^Department of Biological Science, King Abdulaziz University, Jeddah, Saudi Arabia; ^3^International Joint Centre for Biomedical Innovation, Henan University, Kaifeng, China; ^4^Department of Phamocognosy, College of Pharmacy, King Saud University, Riyadh, Saudi Arabia; ^5^Department of Chemistry, King Abdulaziz, University, Jeddah, Saudi Arabia

**Keywords:** Mesoflavibacter zeaxanthinifaciensis, exopolysaccharide, antibacterial, biofilm, lipid peroxidation

## Abstract

Considering the significance of biological and eco-friendly nanomaterials, in the present study, we have synthesized silver nanoparticles from the exopolysaccharide of recently recovered bacterial strain CEES51 from the Red Sea coastal area of Jeddah, Saudi Arabia. 16S ribosomal RNA gene sequencing was used to characterize the isolated bacteria, and it was identified as *Mesoflavibacter zeaxanthinifaciens* and assigned an accession number MH707257.1 GenBank. The bacterial strain is an excellent exopolysaccharide producer and survived at hypersaline (30%) and high-temperature (50°C) conditions. The bacterial exopolysaccharides were employed for the fabrication of silver nanoparticles at room temperature. UV-visible spectrophotometer optimized the synthesized nanoparticles, and their size was determined by Nanophox particle size analyzer and dynamic light scattering. Additionally, the X-ray powder diffraction and Fourier-transform infrared spectroscopy studies also approved its crystalline nature and the involvement of organic functional groups in their formation. The synthesized nanomaterials were tested for their antibacterial and antibiofilm properties against pathogenic microorganisms *Bacillus subtilis* and methicillin-resistant *Staphylococcus aureus*. The antimicrobial property showed time, and dose-dependent response with a maximum of zone inhibition was observed at around 22 and 18 mm at a dose of 50 µg/well against *B. subtilis* and* S. aureus* and a minimum inhibitory concentration of 8 and 10 µg/ml, respectively. Furthermore, the synthesized silver nanoparticles possessed a substantial antibiofilm property and were also found to be biocompatible as depicted by red blood cell lysis assay and their interaction with peripheral blood mononuclear cells and human embryonic kidney 293 cells. Therefore, *Mesoflavibacter zeaxanthinifaciens* is found to be an excellent source for exopolysaccharide synthesis that assists in the silver nanoparticle production.

## Introduction

The occurrence of drug resistance in microbial pathogens and the failure of traditional and conventional medication systems lead to severe health issues worldwide. The uncontrolled use and abuse of antibiotics, along with the slow rate development of new drugs, have increased drug resistance in pathogens (Cassir et al., 2014). Drug resistance occurs due to the unconstrained evolution of rapid and proficient antibiotic resistance gene in the bacterial genome (Reding-Roman et al., 2017) that helps the bacterial cells able to thrive in adverse condition and form the biofilm. The bacterial biofilm is a complex aggregation of the similar bacterial population, which encapsulated in exopolysaccharide (EPS) matrix, and allows the bacteria to gain resistance to drugs than their planktonic forms (Karimi et al., 2015). The number of pathogenic microbes can adhere to the surface of medical devices and persist there for a long time through the development of biofilm, which spread contamination in surroundings and infect human being (Szczotka-Flynn et al., 2009). For example, in the orthopedic field, approximately 6,000 incidents of implant failure are reported annually due to infection by biofilm-forming pathogenic microbes (Teterycz et al., 2010). Owing to the problem of drug resistance, in the last two decades, a number of researchers have emphasized the development of cost-effective drugs with high efficacy (Gould and Bal, 2013; Ventola, 2015; Dong et al., 2016). To overcome the problem of drug resistance, there has been a reappearance in the employment of nanoparticle-based antiseptics having broad-spectrum antimicrobial potential and an extreme lower proclivity to induce drug resistance in pathogenic microorganisms (Fair and Tor, 2014). Nanomaterial-based drugs possess significant advantages over conventional antimicrobial agents. The most important problem with a chemical antimicrobial agent is the expiry and easy development of resistance in pathogenic bacteria. In general, chemical antimicrobial activity mainly depends on the binding of specific surface proteins of the membrane and metabolic factor of microorganism. Since bacteria have progressive drug resistance developments after many generations, antibiotics have become less effective for therapy and limited application in prophylaxis in antimicrobial facilities. Recently, worldwide researchers have focused on variable kind of nanomaterial synthesis and there use as an antimicrobial agent to control infectious diseases and biofilm establishment (Huh and Kwon, 2011). Previously, some metals and their oxide materials like Al_2_O_3_, TiO_2_, ZnO, and silver nanoparticles have been reported for biocidal property against microbes (Behera et al., 2013; Qu et al., 2018). Chemical-based nanoparticles create a health risk and environmental hazards, while the green synthesized nanoparticles are less toxic due to the involvement of a biological functional group (Jia et al., 2017; Strauch et al., 2017). Green nanotechnology proactively influences the designing of nanomaterials and eliminates/minimizes the pollution caused as a result of their production. It is built based on the principles of green chemistry/engineering and focuses on synthesizing nanoscale materials. Synthesized materials stability and impact on the environment are judged by a life cycle approach and should assure less environmental alteration during product synthesis, owing to the use of silver nanoparticles (AgNPs) in various fields including biomedical, biotechnological, and environmental safety purposes. Recently, researchers have used marine microorganisms as a potential biofactory for the bio-fabrication of the specific and unique type of metallic nanomaterial development (Manivasagan et al., 2016). Previous marine organisms like *Alteromonas macleodii* (Mehta et al., 2014), *Yarrowia lipolytica* (Apte et al., 2013), *Ochrobactrum* sp. (Apte et al., 2013), *Streptomyces albidoflavus* (Prakasham et al., 2012), *Pseudomonas aeruginosa* (Ramalingam et al., 2014), and *Stenotrophomonas* sp. (Malhotra et al., 2013), *Sargassum cinereum* (Malhotra et al., 2013) has been exploited for the synthesis of biogenic nanomaterials.

The bacterial strain of *Bacillus subtilis* sometimes may be able to cause severe infection or even fatal disease in human. *B. subtilis* bacteria might cause eye infections and acute conjunctivitis as reported in earlier studies (Wilamowski, 1912; Soule, 1932; Francois, 1934). Wilamowski has published pulmonary infection with hemoptysis and hemorrhagic pleural effusion before death; it was caused by gram-positive, sporulating pseudoanthracis bacilli (Keleman, 1924). A related disease in the lungs, which develops into gangrene, that was created by *B. subtilis* has been reported by Keleman (Bais, 1927). However, these studies are not able to confirm the bacteremia in pulmonary disease caused by bacilli because of the other number of organisms also responsible (Bonino, 1934). Bonino et al. (Hull et al., 1937) have also reported genitourinary tract infection; *B. subtilis* cause it.

Furthermore, a disease like multiple infarcts of the kidneys, pyelonephritis, cystitis, prostatitis, and meningitis that is related to urinary tract infection associated with *B. subtilis* group was confirmed (Shultz, 1938; Weinstein and Colburn, 1950). Still, in some cases, the appearance is doubtful because it is isolated from the primary disease and acting as a secondary invader. Cotton et al. (1987) have investigated 17 cases of bacteremia caused by *B. subtilis* infection in immunosuppressed patient population. With some study, *B. subtilis* has been reported as a food poisoning agent and to cause food-borne gastroenteritis, if a pure culture of bacilli feeding to dogs produced diarrhea and subcutaneous injection of live or dead culture can create the death of the experimental animal within 3 days (Seitz, 1913). Some strains of *B. subtilis* are capable of producing enterotoxin, which is responsible for food poisoning (From et al., 2005). Recently, a *B. subtilis* strain was also recovered from the packaged food products of the Netherlands (Te Giffel et al., 1996).

In recent studies, *Mesoflavibacter zeaxanthinifaciens* was used to extract glutaminase-free ʟ-asparaginase enzyme that was involved in asparagine hydrolysis and played a significant role in cancer cells (Lee et al., 2016). Another study also showed its employment in the extraction of the xylanase enzyme (Oh et al., 2011) that is highly demanding in the bakery industry (Passarinho et al., 2018). *Mesoflavibacter zeaxanthinifaciens* is one of the essential bacteria that produce antibacterial compound during rapid and efficient screening method (Zhao et al., 2018). According to the importance mentioned previously of this marine bacteria, consequently, we employed the same for the biogenic fabrication of effective approach using the EPS from the novel isolate of *Mesoflavibacter zeaxanthinifaciens* as a new reducing and stabilizing agent. A marine bacterial strain CEES51 of *M. zeaxanthinifaciens* was recovered from the coastal region of Jeddah, Red Sea, Saudi Arabia. We have followed the bottom–up approach for silver nanomaterial synthesis without the involvement of reducing reagents or chemicals. Synthesized nanoparticles were characterized by using modern spectroscopy and electron microscopy techniques. Furthermore, the nanoparticles have been applied for their antibacterial and antibiofilm properties against medically important microorganisms. To our best knowledge, with all the possible referencing, we state that it is the first study exploiting the EPS from hypersaline marine bacteria *Mesoflavibacter zeaxanthinifaciens*for for the synthesis of AgNPs and further application as an antibiofilm agent. This work will be a significant impact on emerging green nanoparticles. This synthesis process relies on eco-friendly and cost-effective technologies with great health benefits without environmental hazards.

## Materials Methods

### Chemical and Reagents

Chemicals and culture media were procured from the Himedia Mumbai, India. Crystal violet dye and biochemical assay reagents were also purchased from Himedia Mumbai, India. Specific chemical silver nitrate (AgNO_3_) was purchased from the La Pine Scientific Company, Berkeley, Calif, 94710, Chicago, USA. Bacterial isolates of *Bacillus subtilis* and *Staphylococcus aureus* were obtained from the King Fahad Medical Research Centre, King Abdul Aziz University, Jeddah, Kingdom of Saudi Arabia.

### Isolation of Hypersaline Marine Bacteria and Its Characterization

Marine water samples were collected from 12 m depth of the Red Sea, Jeddah Coastal Region, the Kingdom of Saudi Arabia. For marine bacteria, isolation Zobell media was used in broth as well as plate preparation. Briefly, media were prepared from the following ingredients (grams per liter): peptic digested animal tissue 5.0, sodium chloride 19.45, yeast extract 1.0, ferric citrate 0.1, magnesium chloride 8.80, calcium chloride 1.8, sodium sulfate 3.24, potassium chloride 0.55, sodium bicarbonate 0.16, potassium bromide (KBr) 0.08, stannous chloride 0.34, boric acid 0.02, sodium metasilicate 0.004, ammonium nitrate 0.002, disodium phosphate 0.008, sodium fluorate 0.002, and pH 7.4 adjusted. For solid agar media plate, 20 g/L agar powder was added in media broth before autoclave, after sterilization followed by pouring warm media in Petri plates for solidification at room temperature. From each bottle, marine water (∼100 μl) samples were spread on the Zobell marine media plates that were incubated in an incubator at 30°C for 3 days. After incubation, the plate surface was covered with the bacterial colony, and it was re-streaked on a similar media plate for purification. Purified bacteria colonies were inoculated separately in an Erlenmeyer flask containing Zobell broth medium (50 ml) on an incubator at 120 rpm at optimum incubation conditions. From each flask, 4 ml of incubated broth was collected and centrifuged at 8,000×g for 10 min, and attained supernatant was used for EPS determination by phenol/sulfuric acid method. It was observed that the bacterial isolate number CEES51 exhibited highest EPS-producing bacteria, and it was selected for biochemical and molecular characterization. During biochemical characterization, gram staining, sugar fermentation, and gelatine and starch hydrolysis, indole/methyl red/Voges–Proskauer/citrate utilization test, pigmentation, and flagella motality were observed. Furthermore, the molecular characterization of strain CEES51 was analyzed by 16S ribosomal RNA (rRNA) gene sequencing technology. Partial gene sequencing of 16S rRNA of CEES51 was obtained commercially from Macrogen Incorporation, Seoul South Korea. It was done by using universal primers, 518F and 800R. The forward primer 518F sequence symbol is 5’CCAGCAGCCGCGGTAATACG3’ and 800R reverse primer indicated by 5’TACCAGGGTATCTAATCC3’. Additionally, the sequence data were submitted to the National Center for Biotechnology Information (NCBI) GenBank for obtaining the unique accession number. Maximum homology of this strain was observed by BLASTn online analysis and matched with identified taxonomic information already available on the NCBI (http://www.ncbi.nlm.nih.gov/BLAST) website. The neighbor-joining scheme created the further phylogenetic tree with the help of the MEGA 7.06 program.

### Bacterial Exopolysaccharide Production

For maximum EPS production, a 50-ml Zobell broth was prepared in a 100-ml capacity Erlenmeyer flask and amended in 5 and 10% glucose followed by autoclaving according to standard methods. After autoclaving, each flask was inoculated with 0.1% starter culture of CEES51 strain and incubated at 30°C for 4 days. An aliquot of 25-ml culture was centrifuged at 8,000×g, and the supernatant obtained was exploited for EPS determination. EPS quantification was analyzed by 3:1 volume of chilled ethanol to supernatant at 4°C under overnight incubation conditions. The resulting precipitate was collected and centrifuged at 10,000×g for 30 min. Isolated EPS was dialyzed (basic dialysis bag, Spectra/pour 3, 132720T, USA) overnight at 3.5 KDa against water. After dialysis, it was mixed again with chilled ethanol and nurtured overnight at 4°C. The resulting precipitate was dried at room temperature and stored in a desiccator. For purification, obtained EPS was added to Milli-Q water (5 g/L) and separated with ion exchange chromatography *via* diethylaminoethyl cellulose–Sepharose column (1.6 × 30 cm) at a flow rate of 1 ml/min. Elated fraction (2 ml) was determined by the phenol/sulfuric acid method and stored for the following experiment. Furthermore, percent protein and sugar amount in extracting polysaccharide were determined by the procedure of Lowry et al. (Lowry et al., 1951) and Dubois et al. (Dubois et al., 1956), respectively.

### Synthesis and Characterization of Biogenic AgNPs

For the development of biogenic silver nanoparticles, 1 ml of EPS was collected from CEES51 strain and added to 99 ml aqueous 1 mM solution of AgNO_3_. The sample reaction mixture was then incubated in the dark for variable periods. Unwanted silver ions that were not participating in the reaction were removed by centrifuging the reaction mixture and repeated washing with water. AgNPs obtained were further purified by 12-kD cutoff dialysis bag by resuspending them in 100-ml 2-[4-(2-hydroxyethyl)piperazin-1-yl]ethanesulfonic acid buffer (pH 7.4, 20 mM) and amending with sucrose for maintaining the solution density of 2.5 g/ml for 12 h. The dialysis material was transferred into centrifuge tubes (50 ml) and centrifuged at 10,000 rpm for 1 h at 4°C. The obtained supernatant and pellet, silver ion concentration was determined by inductive coupled plasma emission spectrophotometer (ICPE-900 Shimadzu, Japan) and used for material characterization. Deep brown color that appeared as a result of the incubation was considered to be an indication for the synthesis of silver nanoparticles that was monitored by ultraviolet-visible (UV-Vis) absorption spectra (Smith and Pugh, 1979; Dutta et al., 2012).

#### Ultraviolet-Visible and Nanophox Spectra Examination

The reaction mixture contained both AgNO_3_ and EPS as a capping agent from marine bacteria. Deep brown color that appeared after variable period incubation time due to the bio-reduction of silver ions existing in the reaction mixture was examined by ultraviolet-visible (UV-Vis) spectrophotometer (UV-1800, Shimadzu Japan). The fabrication of AgNPs was confirmed from the peak value obtained between 300- and 600-nm wavelength regions. Moreover, fabricated AgNPs were analyzed by the nanophox particle size analyzer in the aqueous medium (Oves et al., 2018).

##### Characterizations of Particle Size

Dynamic light scattering (DLS) technique was employed to determine the hydrodynamic size distribution of the particles. DLS measurements were analyzed by Malvern Zetasizer Nano-ZS90 (ZEN3590, UK). The average hydrodynamic size of the AgNPs was found to be 35 ± 10 nm and with an excellent polydispersity index (PDI) of 0.2 values in a colloidal suspension with a zeta potential of −30 mV (Ito et al., 2004).

#### Fourier-Transform Infrared Spectroscopy Spectra Analysis of Synthesized Nanomaterials

Biological moieties involved in AgNPs formation were confirmed by Fourier-transform infrared spectroscopy (FTIR) spectra (IR Affinity-1 Shimadzu, Japan). KBr was used as a beam splitter to observe the natural functional group involvement in particle formation. Purified nanomaterial samples were prepared as a disc with KBr crystal, and spectra was recorded in the wavenumber region between 500 and 4,000 cm^−1^ (Carlson et al., 2008).

#### Scanning Electron Microscopy and X-Ray Diffraction Analysis

The morphology of purified AgNPs was scanned by field emission scanning electron microscopy (Hitachi SU6600, Japan) by taking a thin sheet of AgNPs that were mounted on the carbon-coated copper grid. The X-ray diffraction analysis of synthesized biogenic AgNPs was conducted by using Rigaku Miniflex X-ray diffractometer with Cu-Ka radiations 2θ = 0.15406 nm from 30 to 80° with 2 h running time.

### Antibacterial Assay

#### Zone Inhibition Assay

The antibacterial analysis of synthesized AgNPs was investigated against human pathogenic bacteria, *Bacillus subtilis*, and Methicillin-resistant *Staphylococcus aureus* (MRSA). We have developed the fresh culture of test microorganisms in nutrient broth for optimum growth. Initially, 50 ml of nutrient broth was prepared in a conical flask and sterilized at 121°C with 15 psi pressure for 20 min in the autoclave machine. The starting culture (bacterial) was inoculated and allowed to grow at 35 ± 2°C in an incubator for overnight growth to obtain a fresh culture. Concurrently, the nutrient agar plate was prepared according to the standard method of sterilization for antimicrobial activity (Wiegand et al., 2008).

The previously obtained bacterial culture was spread on nutrient agar plates with the support of sterilized cotton wad. After a 10-min incubation, plates were dried, and a 5-mm well of 100-µl capacity was prepared in two positions in plate solidified media with the help of sterilized borer. Furthermore, the bottom of each well was sealed by liquefied agar to avoid the leakage of loaded compounds. Next, 10-µl suspensions containing 5- to 50-µg AgNPs were loaded in each well and incubated at 35 ± 2°C overnight. After incubation, a zone of inhibition of bacterial growth that appeared around the well as a result of the antimicrobial action of AgNPs was recorded (Rauf et al., 2017).

#### Minimum Inhibitory Concentration and Minimum Bactericidal Concentration Determination

*Bacillus subtilis* and MRSA bacterial culture were diluted with growth-supporting nutrient broth to adjust the concentration up to 1 × 10^6^ colony-forming unit (CFU)/ml. Then, the stock suspension solution of test nanomaterials was prepared with 1:1 dilution of bacteria that was prepared from a new culture and nutrient broth in test tubes. Furthermore, all dilutions of test compounds were inoculated with an equal volume of fresh culture. Positive and negative controls were also prepared separately without compound, and without compound and organisms, respectively, and incubated at 35 ± 2°C overnight. Turbidity was checked as an indicator of the growth of the bacterial organism in the presence of AgNPs. The least amount dose of AgNP that inhibited the growth of the organism indicated the minimum inhibitory concentration (Rauf et al., 2018).

#### Antibiofilm Activity Determination

##### 2,3-Bis(2-methoxy-4-nitro-5-sulfophenyl)-2H-tetrazolium-5-carbox-anilide Biofilm Assay

The antibiofilm nature of synthesized nanoparticles was established employing 2,3-Bis(2-methoxy-4-nitro-5-sulfophenyl)-2H-tetrazolium-5-carbox-anilide (XTT) assay against MRSA and *B. subtilis* biofilms (Kulshrestha et al., 2014; Xu et al., 2016). Briefly, postmature biofilms, the plate was carefully washed with phosphate-buffered saline (PBS) to eradicate nonadherent cells. Afterward, the established biofilm was incubated with increasing concentration of AgNPs and incubated at 37°C for 48 h. After a stipulating time interval, 2,3-Bis(2-methoxy-4-Nitro-5-sulfophenyl)-5-([phenylamino] carbonyl)-2H-tetrazolium hydroxide (XTT solution) was amended, and final concentration becomes 5 mg/ml. The prepared solution of the XTT was filtered by employing a 0.22-mm pore-size filter and kept at −80°C. Menadione (0.4 mM) solution was primed and filtered instantly just earlier the commencement of the examination. The adherent bacterial cells were eroded with buffer (PBS, 200 μl), followed by the XTT solution, and 2 μl of menadione into every well. After a 4-h incubation at 37°C in the dark, the obtained solution was resuspended and shifted to a new plate and assessed at 490 nm by a microtitre plate reader (BIORAD). Each set of trials was run in triplicate, and the data were communicated as mean ± SD. Further crystal violet staining was also used to assess the biofilm formation. After incubation, each well was washed multiple times with PBS and dried on the paper towel in an inverted position. Further biofilm establishment was quantified by staining with crystal violet (0.1%) and incubated for 10 min at room temperature, further washed thrice with Milli-Q water and dried carefully.

#### Colony-Forming Unit Determination to Estimate the Bacterial Susceptibility Against AgNPs

The overnight grew a bacterial culture of MRSA, and *B. subtilis* strains were sub-distributed into six test tubes (cell density ∼10^6^–10^7^ cells/ml). Furthermore, a 100-μl aliquot of AgNP solution from the stock solution (10 mg/ml) followed by a final stock of ampicillin solution (100 μg/ml), for negative control (media only) and positive control (100 μl PBS + culture), was distributed to consistent tubes and incubated for the next 4 h at 37°C. After that, 100-μl aliquot cultures from each control and treated cells were transferred to be plated in duplicate at 1:1 and 1:10 dilutions onto the brain heart infusion agar plates and transferred to an incubator chamber for 24 h at 37°C. After the specific incubation time interval, the colony forming unit at variable dilutions was calculated and represented as log_10_ colony-forming unit per milliliter, and the colony counts from two separate experiments were averaged from each organism (Rauf et al., 2017; Oves et al., 2018).

#### Bacteria–Nanocrystals Interaction as Exposed by Electron Microscopy

The MRSA and *B. subtilis* bacterial isolates were exposed to AgNPs for 4 h, at 37°C, with consistent agitation in an incubator. Further achieved cell suspension was eroded three times in brain heart infusion medium to evacuate unbound or approximately related nanoparticles. The bacterial cells that interacted with AgNPs at specified time interim were arranged and imaged by scanning electron microscopy (SEM). Moreover, the microbes were also imaged by transmission electron microscopy (TEM). For TEM, the bacterial strains (∼10^7^ CFU) were stable with 2% glutaraldehyde within PBS and consequently presented to osmium tetroxide (1%) in aqueous solution, for 8 h. The samples were filtered through a 0.22-μm pore estimate channel (Millipore) and drained with acetone and, in this manner, with liquid CO_2_ in an essential point drying contraption (SPI, USA). Channel paper areas were metalized with gold, by sputter covering, and scanned with a JEOL JSM-6390 LV (Xie et al., 2011).

#### Bacterial Cell Viability Assay Employing SYTO9 and Propidium Iodide Dyes

To judge the viability of bacterial cells upon treatment with the biogenic silver nanoparticles, an SYTO9-PI (Invitrogen, CA) bacterial viability kit was used. An aliquot (10 μl) of a solution consisting of an equal amount of SYTO9 and PI solution was amended into the well of treated cells in each plate and incubated for 15 min in the dark at room temperature. After the incubation, cells were eroded with cold, sterile PBS and fixed on a glass slide. The cells were visualized by fluorescence microscope (Zeiss, USA) (Ahmad et al., 2015).

#### Intracellular Reactive Oxygen Species Production by AgNPs Inside Bacterial Cells

The bacterial cells whenever exposed to the AgNP then reactive oxygen species (ROS) were generated in the intracellular space. It was detected by the fluorescent probe 2,7-dichlorofluorescein diacetate (DCFH-DA) because these dyes passively diffuse into the membrane of the bacterial cell.

If the dye internalized in the bacterial cell, it becomes deactivated catalyzed by esterases enzyme to form a non-fluorescent 2,7-dichlorofluorescein (DCFH). The ROS formation in the cells reacts with DCFH to form the fluorescent 2,7-dichlorofluorescein (DCF) product that trapped inside the cell and become fluorescent. Concisely, both the bacteria MRSA and *B. subtilis* (culture 10^6^ CFU/ml) treated with AgNPs at their subminimum inhibitory concentrations (MIC) for 1 h were eroded three times with uncultured media. DCFH-DA dropwise added and mixed with the bacterial cells and incubated at 37°C for 30 min in a shaking incubator. The cells were pelleted down and washed with Milli-Q water to remove the unbound DCFH. Both control and experimental-treated bacterial cells were visualized under a fluorescence microscope (at 20×, magnification scale Zeiss model, United States). Here noted, the fluorescence intensity is directly proportional to the volume of ROS generated (Williams et al., 1998).

#### Determination of Cellular Respiration

The stationary phase has grown a bacterial culture amended with different concentrations of biogenic AgNPs and incubated at 37°C for 4 h in a shaker incubator at 120 rpm. After proper incubation, treated cultures (100 ml) wEre centrifuged at 5,000×g for 30 min, and the pellet was transferred in 10 ml of sterile normal saline solution and used for performing the assays. The reaction blend contained 2-ml AgNPs-cell slurry and monopotassium phosphate buffer (pH 7.0, 12.5 mM). The reaction was started by adding 50-ml glucose (1 M) as an electron donor and amend with 1% of 2,3,5-triphenyltetrazolium chloride (TTC) solution and incubated at room temperature for 2 h. TTC gets reduced to 2,3,5-triphenyltetrazolium Formosan (TTF) solution, thereby resulting in the formation of a dark color. All the samples from the reaction mixture were centrifuged at 8,000×g for 20 min, and the pellets were obtained by shaking with 4 ml of methanol for 10 min. The absorbance of the red supernatant obtained was measured spectrophotometrically at 485 nm (Wang et al., 2012). For calculation, a standard curve was prepared from the varying concentrations of the TTF prepared in methanol, which had a specific value for E485 of concentration 27.5 mM^−1^cm^−1^. The reduction rate of O_2_/TTC was indicated in nanomoles of O_2_ per TTF per minute per milligram (dry weight) of cells (Delbarre-Ladrat et al., 2014).

#### Lipid Peroxidation of Membrane

Bacterial cell lysis was determined by observing membrane damage *via* lipid peroxidation assay by using malondialdehyde (MDA) formation as an indicator. MDA formation was calculated by thiobarbituric acid (TBA)-mediated reaction that forms MDA-TBA pink color adducts (Dutta et al., 2012). According to this method, AgNP-cell slurry (2 ml) was mixed with 10% w/v trichloroacetic acid (4 ml), and the resulting reaction blend was centrifuged at 10,000×g for 10 min. The obtained precipitate was removed. The supernatant was exploited for subsequent reactions. Freshly prepared TBA solution (0.67% w/v) was added to the supernatant, and the reaction sample was incubated in a water bath at 80°C for 10 min and cooled to room temperature. The absorbance was spectrophotometrically analyzed at 532 nm. The concentrations of MDA formed were analyzed by interpolating on the standard curve available for MDA-TBA complex, which was 49.5 mM^−1^ cm^−1^ for E532 (Sigma Chemical Co). Lipid peroxidation was represented in nanomoles of MDA per milligram (dry weight) of cells (Ahmad et al., 2015).

#### Cytotoxicity Assays

##### In Vitro Erythrocyte Lysis Test

For future potential biomedical applications, the cytotoxicity of the in-house AgNPs was studied against red blood cells (RBCs), peripheral blood mononuclear cells (PBMCs), and human embryonic kidney (HEK)-231 cell line. Initially, *in vitro* erythrocyte lysis experiment was performed. In this experiment, nanomaterials interact with erythrocyte that caused membrane leakage and disruption that released hemoglobin; this hemoglobin was measured. Briefly, fresh blood was obtained from a healthy rabbit, and it was collected with an anticoagulant solution (ethylenediaminetetraacetic acid) and centrifuged at 8,000 rpm for 10 min at 4ºC. After centrifugation, obtained buffy coat was discarded, and erythrocyte was collected and washed with diluted isotonic buffer (20 mM PBS) and prepared with 50% hematocrit. The extent of hemolysis was analyzed by incubating the RBC suspension with different concentration of AgNPs at 37ºC for 1 h. All samples from the incubated solutions were centrifuged at 10,000 rpm for 10 min, and obtained supernatant was collected and measured with an absorption at ƛ_max_ 490 nm by UV-Vis spectroscopy.

The following equation analyzed the % hemolysis:

%Haemolysis=[{AbsT−AbsC}/Abs(100%)−AbsC]×100

In the discussed equation, Abs_T_ has represented the absorbance of the supernatant from samples incubated with AgNPs, Abs_C_ is the absorbance of the supernatant from control (PBS). Abs_(100%)_ represents the absorbance in the presence of 0.1% Triton X-100 as a positive control. All experiments are performed in triplicate and represented as SD.

##### Thiazolyl Blue Tetrazolium Bromide Analysis

The thiazolyl blue tetrazolium bromide (MTT) assay was performed to assess the viability of PBMCs and HEK-231 after AgNP exposure. MTT is a yellow dye that gets converted into formazan, by the activity of the mitochondrial dehydrogenase enzyme. Briefly, 10^4^ cells per well were seeded in 96-well plates and allowed to incubate overnight. Next morning, the medium was aspirated, and the cells were incubated with varying concentration of ZnO-NRs at 37°C for 24 h followed by 4 h with MTT dye (5 mg/ml in PBS). Next day, the reaction mixture was aspirated, and the resulting formazan crystals were dissolved by inoculating 200 μl of dimethyl sulfoxide followed by observing absorbance at 490 nm on a Genetix580 microplate reader (USA). Untreated sets served as control that was performed simultaneously under identical conditions. Finally, OD values of culture were converted into percentage viability by using the following formula (Rauf et al., 2017; Oves et al., 2018):

$$Cell\;viability\;(\%)=\left[\frac{OD_{Sample}}{OD_{Control}}\right]\times 100$$

#### Statistical Analysis

Statistical calculations were performed with the help of Graph-Pad Prism version 6.0, GraphPad software Inc San Diego, California, USA. All result values were expressed as the mean ± SD. Data were analyzed using both one-way analysis of variance (ANOVA) and two-way ANOVA to assess the differences among various groups. Significance was indicated as *** for *P* ≤ 0.001, ** for *P* ≤ 0.01, and * for *P* ≤ 0.05. The student T-test was used to measure biochemical differences between treatment groups and differences with *P* ≤ 0.05 considered as significant.

## Results

### Bacteria Isolation and Characterization

**Figure 1:**
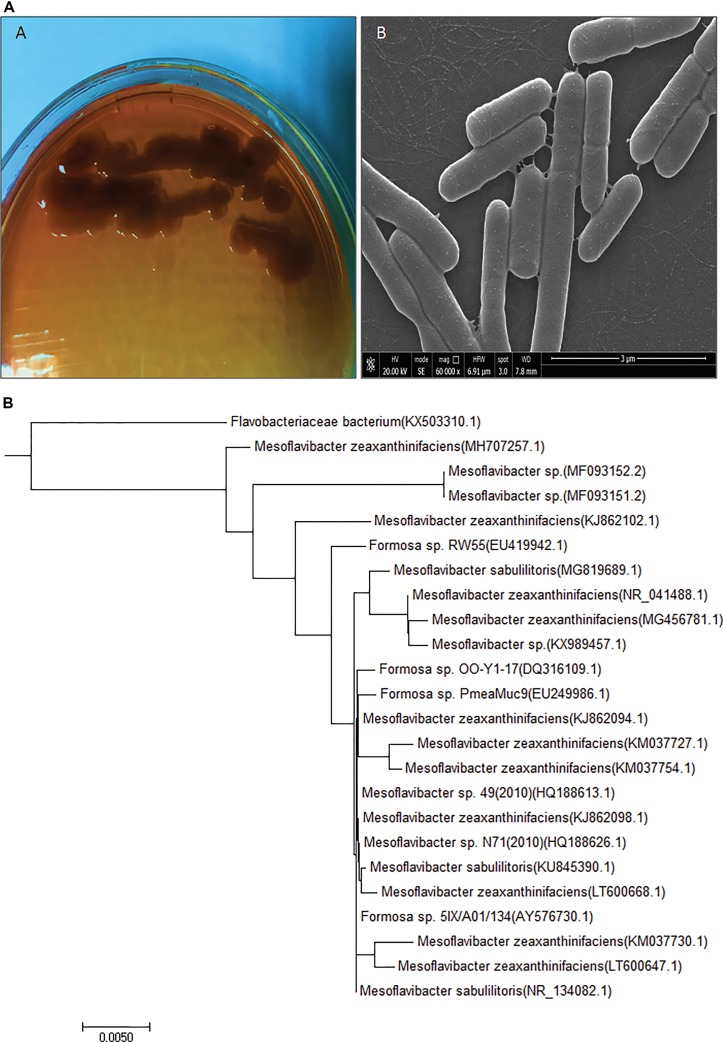
Newly isolated exopolysaccharide producing and hypersaline bacterial strain information **(A)** *Mesoflavibacter zeaxanthinifacien* purified culture on solid agar Zobell’s medium plate and scanning electron microscopy image. **(B)** Phylogenetic tree of *Mesoflavibacter zeaxanthinifacien* created by a neighbor-joining scheme based on accession number of gene sequences.

For potential EPS producing bacterial strain isolation, marine water samples were collected from the 12-m deep Red Sea water near Jeddah coastal area (21°32’N, 39°10’E), Saudi Arabia. Among 50 bacterial isolates, CEES51 were screened out by EPS production. Bacterial isolate CEES51 showed the excellent EPS production capability in hypersaline condition. Furthermore, it was selected for biochemical characterization, molecular identification, and phylogenetic studies. Bacterial cells of the strain CEES51 were found to be gram-negative, aerobic, rod shaped, and non-spore forming (**Figure 1A**), and the biochemical detail is shown in the **Table 1**. CEES51 was able to tolerate salinity up to 20 g/L in growing media at 20 to 45°C. The 16S rRNA gene sequencing was completed with selected bacterial strains, and their identity was searched according to sequence maximum likelihood by using basic local alignment search tool analysis. Molecular identification of CEES51 strain was based on 16S rRNA gene sequence; it was submitted to the NCBI database and assigned an accession number MH707257.1. Furthermore, the phylogenetic tree of *Mesoflavibacter zeaxanthinifaciens* was created by the neighbor joining method by MEGA 7.06 program as depicted in **Figure 1B**.

**Table 1:** Biochemical characteristics of novel bacterial isolate *Mesoflavibacter zeaxanthinifaciens* CEES51.

Characteristics	Features
Morphology	Rod
Gram staining	Negative
Flagella	Motile
Pigmentation	Dark brown
Salinity	10–30%
Temperature	20–50°C
Optimum	35
Catalase	+
Oxidase	−
Sugar fermentation	
D-glucose	+
Lactose	+
Maltose	+
Sucrose	−
Hydrolysis of starch	−
Hydrolysis of gelatin	−
Tween 20	+
Indole	−
Methyl red (MR)	−
Voges–Proskauer (VP)	−
Citrate	−
Urea	−
Presumptive	*Mesoflavibacter*sp.

### Bacterial Exopolysaccharide Production

Among the 55 bacterial isolates from marine water samples, bacterial isolate CESS51 produced the highest EPS and was selected for further studies. A total 32.5 g/L of EPS was formed in growing media at 10% glucose concentration after a 72-h incubation. Monosaccharide composition analysis revealed that it contains ∼45.6% galacturonic acid and ∼27.5% mannose as well as ∼18.5% rhamnose and ∼8.4% glucose.

#### Synthesis and Characterization of Biogenic AgNPs

**Figure 2:**
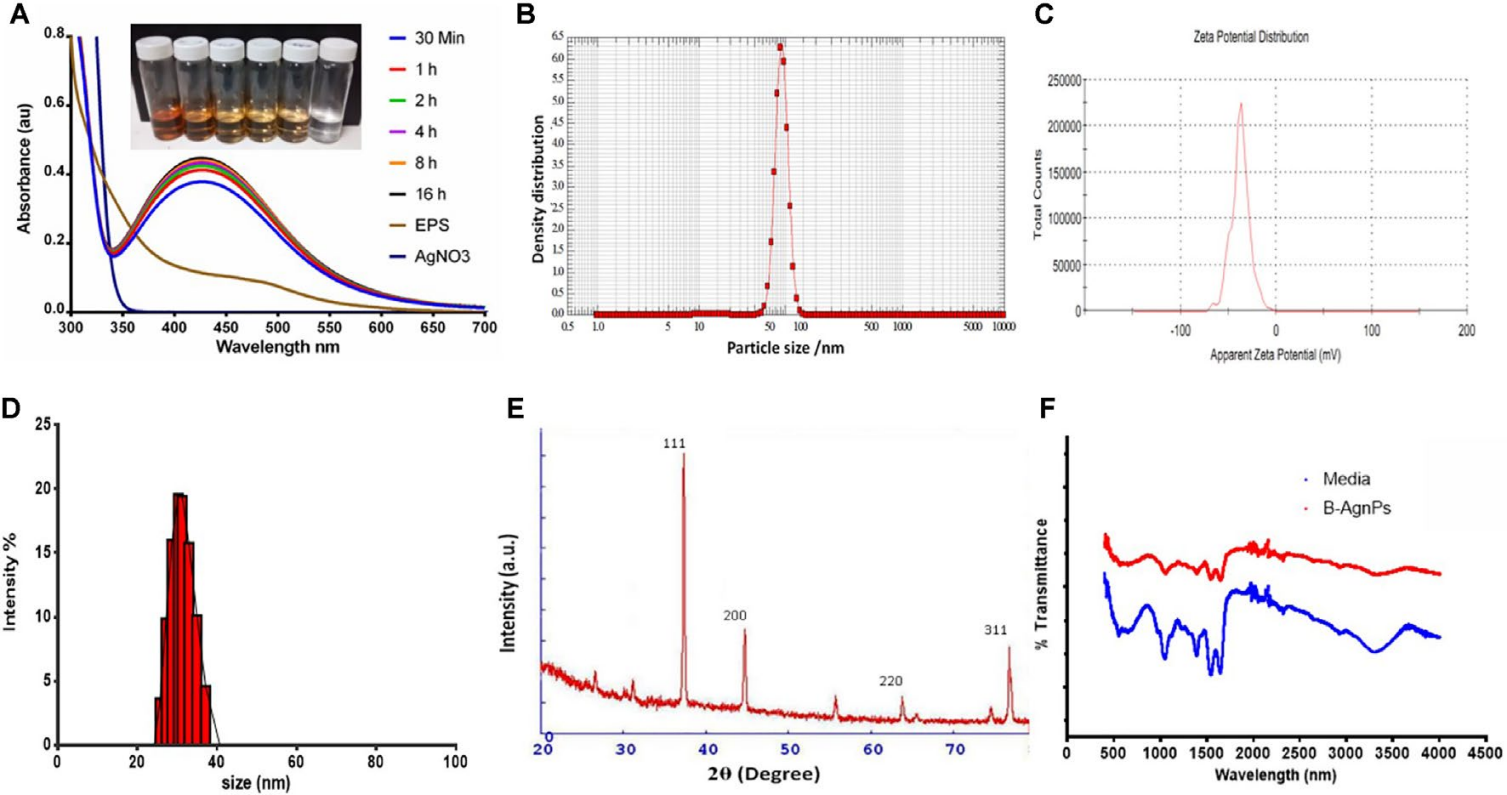
Characterization of biosynthesized AgNPs from the exopolysaccharide (EPS) of *M. zeaxanthinifacien*. **(A)** Ultraviolet-visible (UV-Vis) spectroscopy data displaying a sharp peak between 415 to 430 nm region for the synthesis of B-AgNPs employing bacterial EPS (inlet represents the change in color due to AgNPs formation), **(B)** particle size analysis by Nanophox particle size analyzer, **(C)** particle zeta potential observed −30 mV with excellent polydispersity index, **(D)** Dynamic light scattering (DLS) analysis for hydrodynamic particle size distribution intensity range, **(E)** X-ray powder diffraction (XRD) analysis graph revealed the highest peak matching with crystallite of silver particles, **(F)** Fourier-transform infrared spectroscopy (FTIR) spectra displaying the participation of organic functional group in particles development.

**Figure 3:**
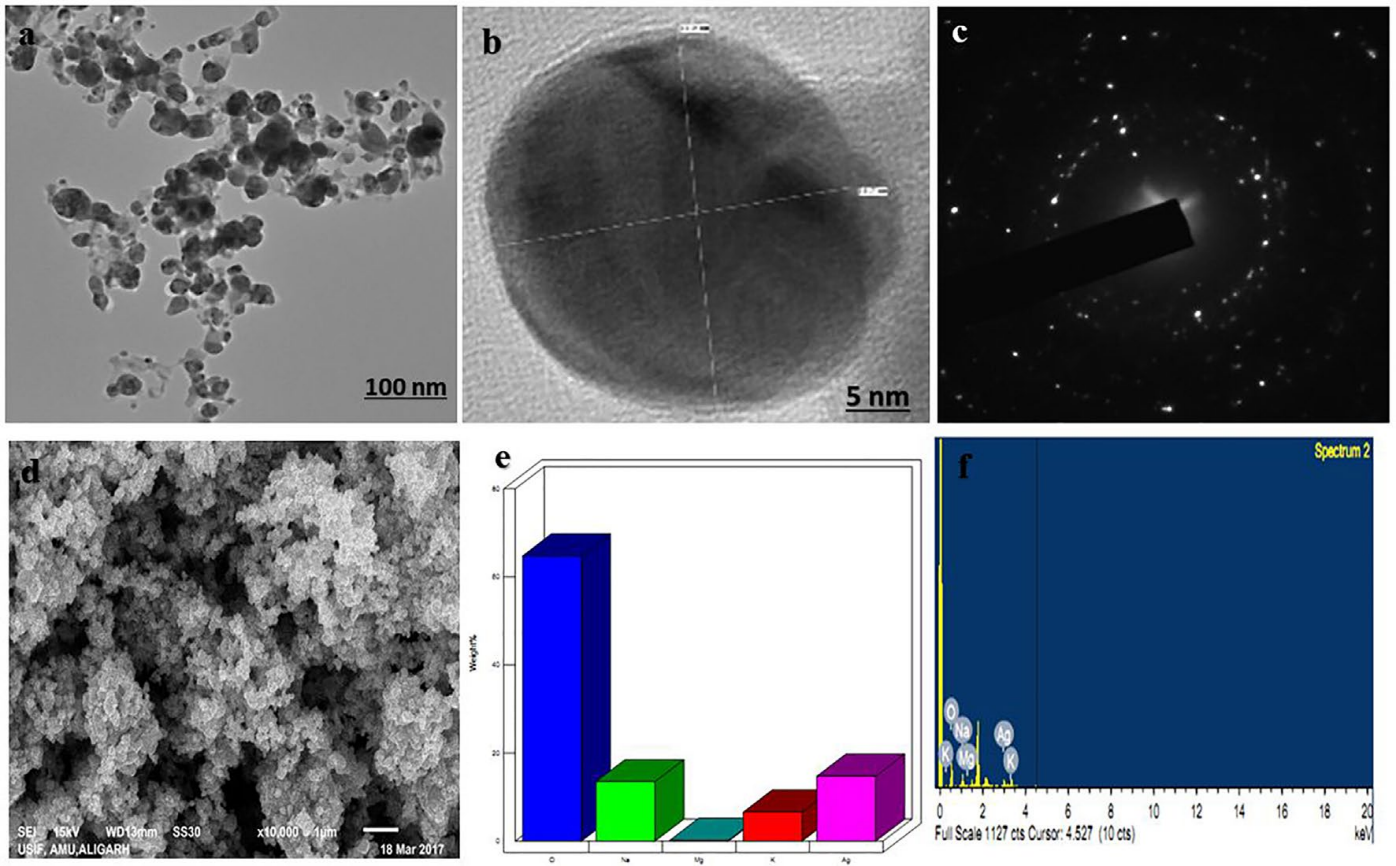
Electron microscopic analysis of as-synthesized b-AgNPs. **(A** and **B)** Transmission electron microscopy (TEM) of AgNPs represents the structure of NPs at 100 nm scale and **(B)** at 5 nm scale. **(C)** The TEM image of the electron diffraction. **(D)** SEM micrograph depicts the NPs microstructure. **(E** and **F)** Energy dispersive X-ray (EDX)-spectrum is representing components of the NPs.

**Figure 4:**
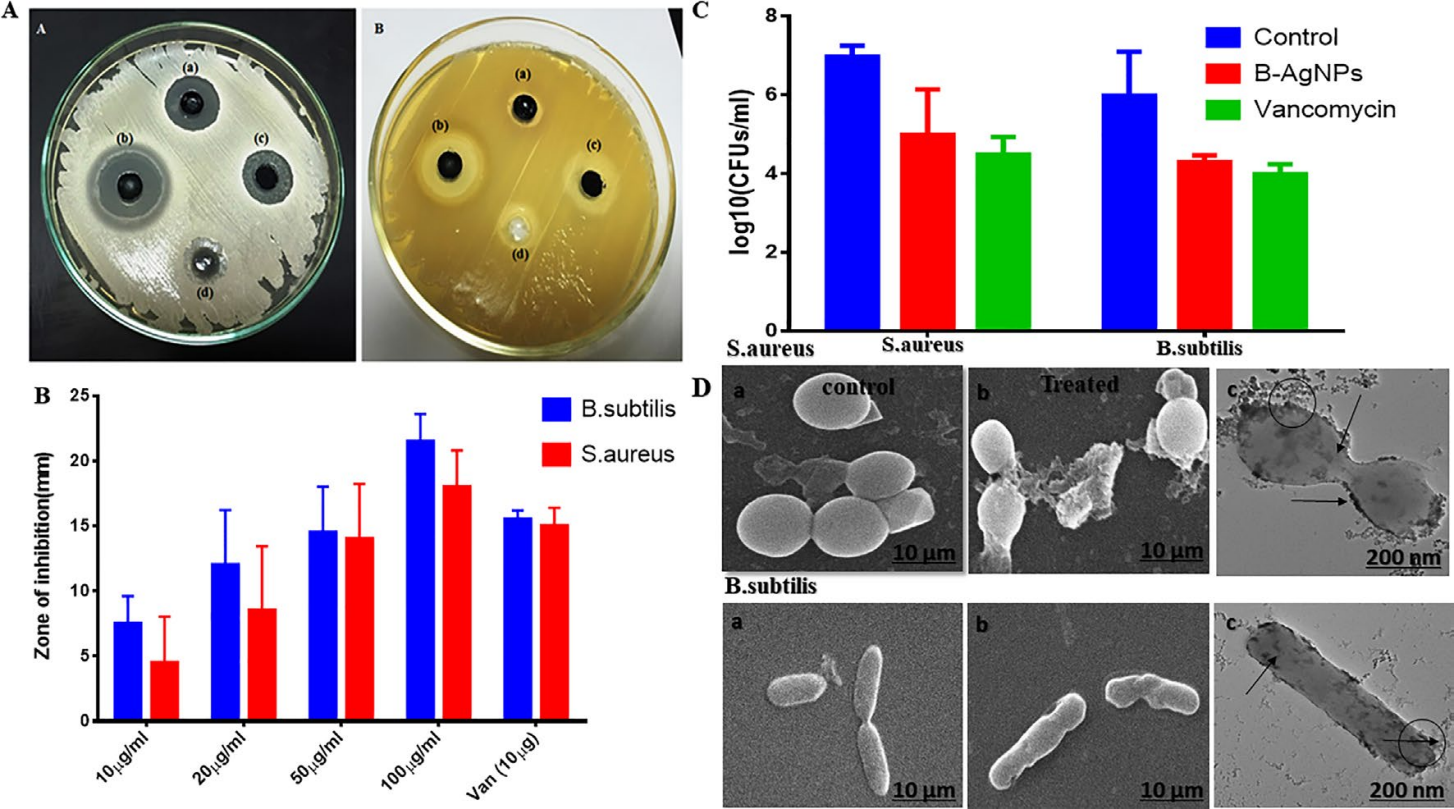
Antibacterial activity of as-synthesized biogenic silver nanoparticles (B-AgNPs). **(A** and **B)** Zone of inhibition as a measure to establish the antibacterial potential of B-AgNPs at varying concentrations against methicillin-resistant *Staphylococcus aureus* (MRSA) and *B. subtilis* strains. **(C)** Colony-forming unit (CFU) counts as residual MRSA and *B. subtilis* surviving after exposure to B-AgNPs. **(D)** Scanning electron microscopy (SEM) micrograph depicting the interaction of B-AgNPs with MRSA and *B. subtilis* strains at their subminimum inhibitory concentrations (sub-MIC).

EPS produced by strain CEES51 of *Mesoflavibacter zeaxanthinifaciens* was applied on the bio-fabrication of silver nanoparticles from the 0.1 mM of silver nitrate in aqueous solution. The addition of 1% bacterial-based EPS in 100 ml of AgNO_3_ solution that was incubated overnight at room temperature converted into a dark brown color, which indicates the development of nanoparticles in the reaction blend (**Figure 4**). The confirmation of nanoparticle formation in the reaction mixture, it was analyzed by the characteristic features through surface plasmon resonance, it was measured by peak spectra at 421 nm that was analyzed from the UV-Vis spectrophotometer (**Figure 2A**). Furthermore, the reaction efficiency of the nanoparticles from the reaction mixture filter from the 12-kD cutoff dialysis bag were purified and checked by elemental analysis using inductively coupled plasma mass spectrometry (data not shown). After overnight incubation, 1% polysaccharide in aqueous silver nitrate had efficiently converted 80% silver into silver nanoparticles through a bottom–up approach. Nanoparticle sizes that ranged between 10 and 35 nm in the reaction mixture were confirmed by the Nanophox particle size analyzer and DLS (**Figures 2B, D**), and these were found to have PDI of 0.2 with a zeta potential of −30 mV (**Figure 2C**). However, the crystalline nature of the nanoparticles was measured by X-ray powder diffraction (XRD) analysis, and peak appearance in 111, 200, 220, and 311 has a good correlation with monophasic silver nanoparticle formation (**Figure 2E**). FTIR analysis confirmed the involvement of the biological functional group in the biological synthesis of AgNPs. To understand the biological groups in the capping of nanoparticles, FTIR machine was run in the range from 0 to 4,000 cm^−1^ (**Figure 2F**). Spectrum analysis revealed the presence of a different peak at different locations, which indicates that different biological groups are involved in nanoparticle formation. More prevalent peaks arise at 3,410 cm^−2^, which indicates the presence of hydroxyl and amine group, peak at 2,920 cm^−1^ for alkyl and CHO group involvement; while at 1,580, 1,640, and 1,650 cm^−1^ indicate the availability of O = C=O of carboxylate and C = O of amide group; and at 1,364 cm^−1^ also indicate the COO2 anion group involved in capping of present nanomaterials. In this study, a typical absorption spectra peak appeared between 1,600 and 600 cm^−1^, which points to the bacterial polysaccharide and functional group from the β-glycosidic linkage it is connected with and sugar moieties spectra peak that arises at 850 cm^−1^. Furthermore, the morphology of synthesized nanomaterials was confirmed by field emission scanning electron microscopy analysis. Field emission scanning electron microscopy and TEM image indicates the hexagonal shape of synthesized silver nanoparticles and the size of most of the particles <40 nm (**Figure 3**).

### Antibacterial and Antibiofilm Assay

Antimicrobial activity of biogenic silver nanoparticles was tested against two medically important organisms *Bacillus subtilis* (gram-positive) and MRSA (gram-positive). When the variable concentration of nanoparticles was applied for well diffusion assay, a zone of inhibition pattern increased significantly with an increased concentration in both cases. A zone of inhibition with a minimum size of 12 ± 2 and 10 ± 2 mm diameter and a maximum of 18 ± 2 and 22 ± 2 mm diameter was observed at 12.5 and 100 µg/well of nanomaterials that were applied against *B. subtilis* and MRSA, respectively (**Figures 4A, B**).

The MIC and minimum bactericidal concentration (MBC) of synthesized nanomaterials were determined in liquid culture media at different concentration. During the investigation, both MIC and MBC were determined through optical density measurement and parallel spread culture on nutrient agar plate count method. The MIC value was taken by average of three of the lowest concentrations that affects the typical growth pattern of the bacterial strain. The MIC values were found to be around 8 and 10 µg/ml for study bacteria, respectively, whereas MBC values were 12.5 and 50 µg/ml of silver nanoparticles against MRSA and *B. subtilis*, respectively.

Furthermore, the effect of synthesized silver nanomaterials in fixed amounts (50 µg/ml) on *Bacillus subtilis* cell morphology as a function of time has been analyzed, and parallel bacterial count was also observed on the solid agar nutrient plate (**Figure 4C**). Furthermore, there was a substantial drop in the bacterial count [*p*-value < 0.01 (**)] of both the studied bacterial strains in the set of those treated with AgNPs as compared with that of the untreated control and also showed response satisfactorily when compared with ampicillin treatment. The observation concords with the zone of inhibition assay. Nevertheless, it is valued revealing that a significant fall in CFU was evident in both the cases when compared to the positive control group of bacteria (**Figure 4C**).

Finally, we checked the effect of nanoparticles after 12-h incubation and observed the disturbed bacterial cell morphology in scanning electron microscopy analysis while plate count also devoid of any viable growth as shown in **Figure 4D**.

**Figure 5:**
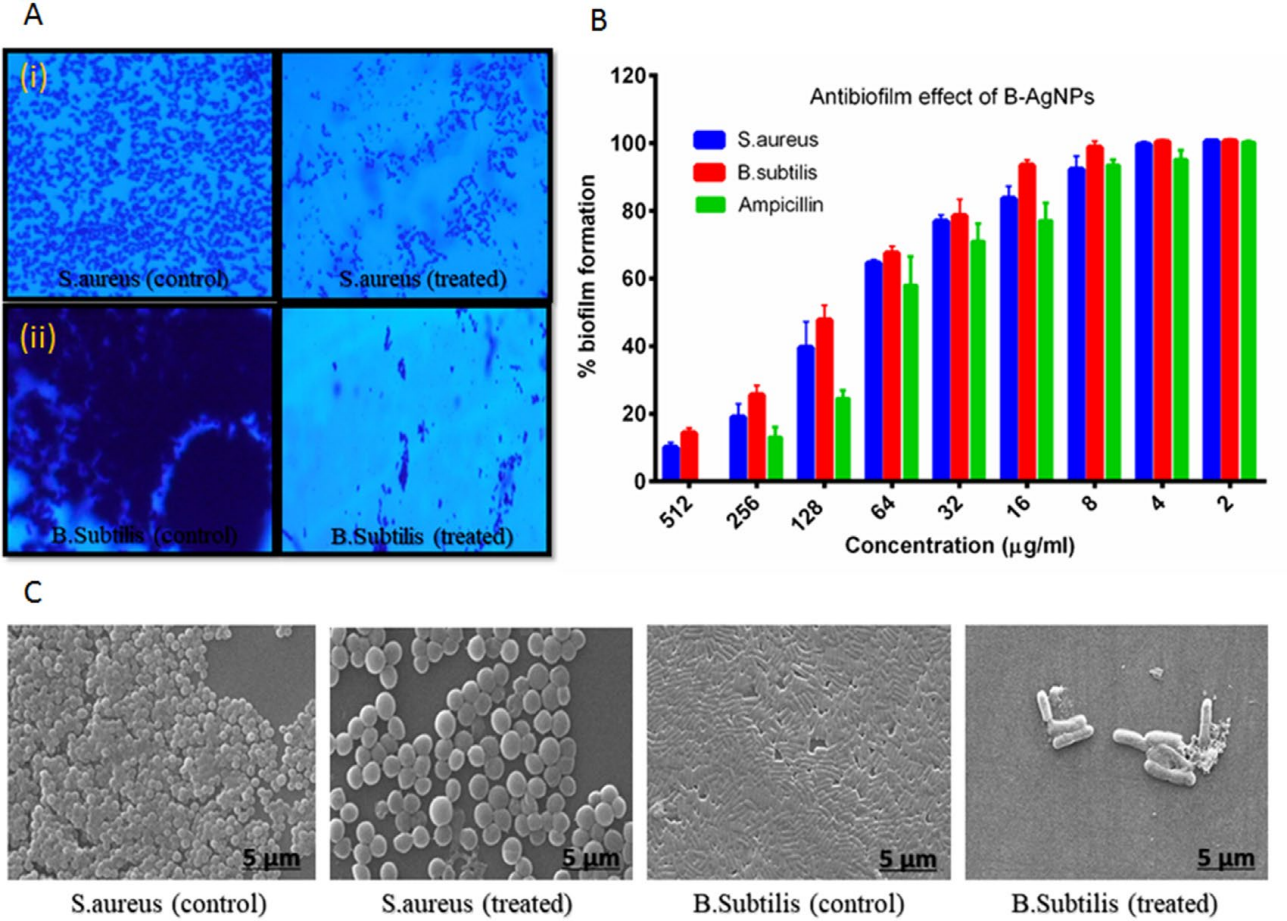
Biosynthesized AgNPs activity against biofilm formation **(A)** revealed the effect of the material on biofilm formation by crystal violet staining (i) MRSA and (ii) *B. subtilis*. **(B)** Observed excellent effect of the content on biofilm formation as confirmed by XTT assay. **(C)** SEM image represent the impact of materials on biofilm formation.

Next, antibiofilm nature of synthesized silver nanomaterials has been investigated against gram-positive bacteria MRSA and gram-negative *B. subtilis.* Biofilm formation depends on multiple factors like extracellular binding proteins and extracellular polysaccharide, adhesion factors, and autolysins. The biofilm inhibitory action of silver nanoparticles arises due to the interference with the internal metabolic system that inhibits the EPS production and protein factor production, which is responsible for biofilm formation. In the present study, MRSA and *B. subtilis* were cultured in 96-well plates and treated with varying doses of silver nanoparticles. Crystal violet staining and XTT assay confirmed the significant inhibitory effect on biofilm formation, which was further explored by scanning electron microscopy, as represented in **Figure 5**. Application of silver nanoparticles at varying concentrations demonstrated a significant reduction (>90%) in the biofilm formed in both the studied strains, as shown in **Figure 5B**. The findings of the crystal violet and the XTT assay were further confirmed by SEM, and reduced microcolonies were observed in nanoparticle-treated SEM micrograph (**Figure 5C**) as compared with the untreated positive control. Further staining with PI and SYTO-9 dye after the treatment with AgNPs at their sub-MIC concentrations showed the presence of red-colored fluorescence that is developed due to the DNA breakage resulting in more staining of PI, thus justifying our above of killing efficiency of our *in situ* synthesized silver nanoparticles (**Figure 1S** supplementary dataset). Moreover, the TEM analysis of bacterial cell data justifies the ability of silver nanoparticles to adhere to its surface and gets into the bacterial cells and thereby damaging the cells (**Figure 5D**).

**Figure 6:**
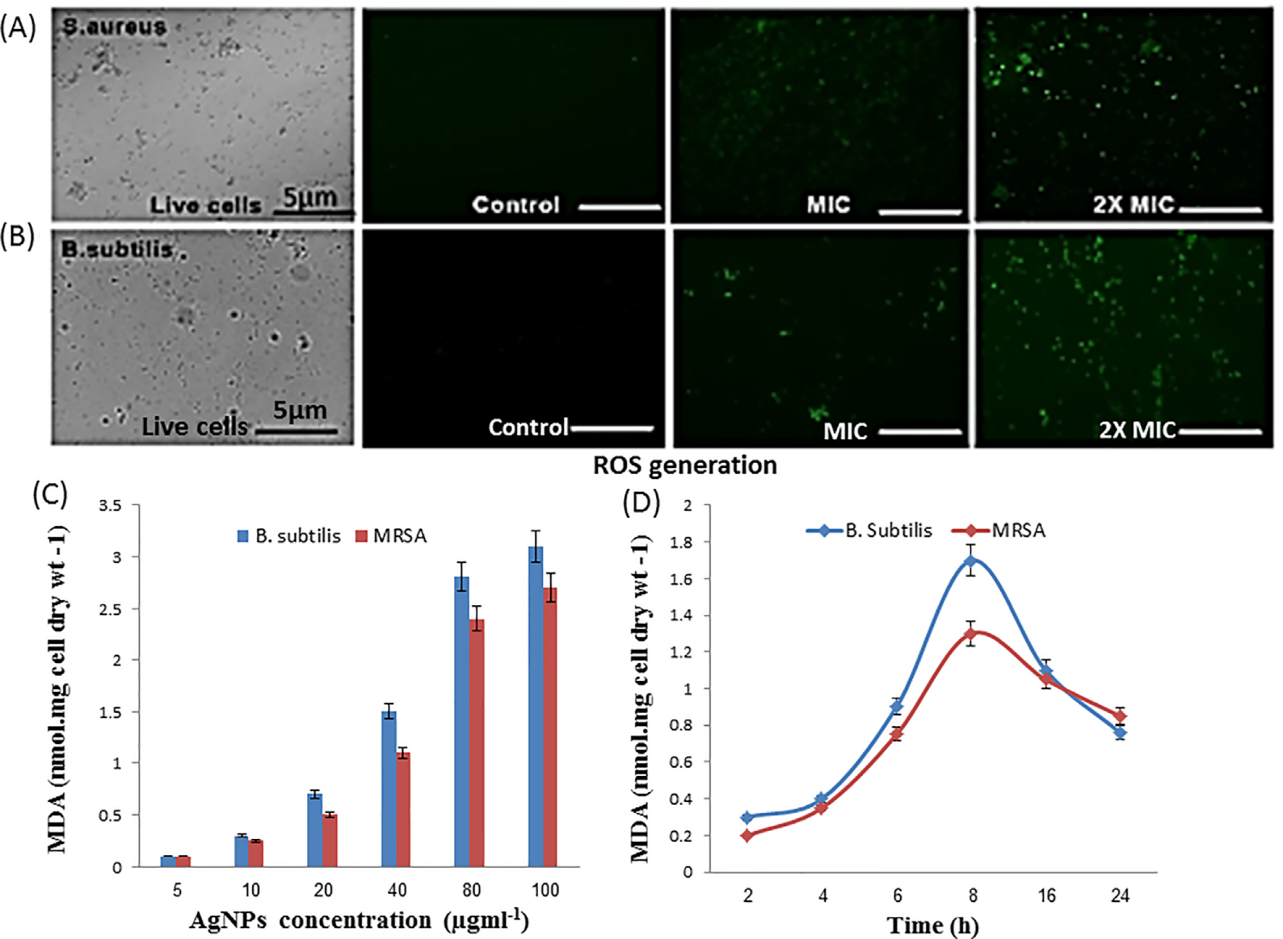
Antibacterial activity of as-synthesized B-AgNPs formulations. **(A)** Level of reactive oxygen species (ROS) production by MRSA. **(B)** *B. subtilis* after treatment with AgNPs at different concentrations as depicted by fluorescence microscopy. **(B)** After a treatment mechanism action of nanomaterial against in microorganisms. **(C)** Malondialdehyde (MDA) production when bacteria treated different dose of nanomaterials and **(D)** at a fixed dose (50 µg/ml) and different incubation time.

Interaction of silver nanoparticles with bacterial membrane proteins resulted in decreased cellular respiration and induced lipid peroxidation. Potential redox properties of amino acid Ag+ interaction in the biological system is due to the possible sequential oxidation reduction and production of ROS like hydrogen peroxide and hydroxyl radical through lipid peroxidation that is indicated by MDA formation and DCFH-DA dye-based ROS generation results obtained from fluorescence microscopy (**Figures 6A, B**). The level of MDA in both treated cultures of bacteria increases with increasing concentration of nanoparticles and incubation time (**Figures 6C, D**). *B. subtilis* and MRSA bacterial culture treated with the different dose of silver nanoparticles showed production of MDA (3.1 and 2.7 nm/cell dry wt). at 100 µg/ml AgNPs in growing media, respectively. Both bacterial cultures supplemented with 50 µg/ml AgNPs and incubated for different time intervals; then, MDA production increased, according to cell activity; highest 1.7 and 1.3 nm/cell dry weight of MDA was observed in the stationary phase of bacteria after 8 h incubation in both bacterial cultures, respectively (**Figure 6D**). Here, MDA production results indicate that increasing concentration of AgNP and incubation time enhance the free radical production in media.

#### Cytotoxicity Assays

**Figure 7:**
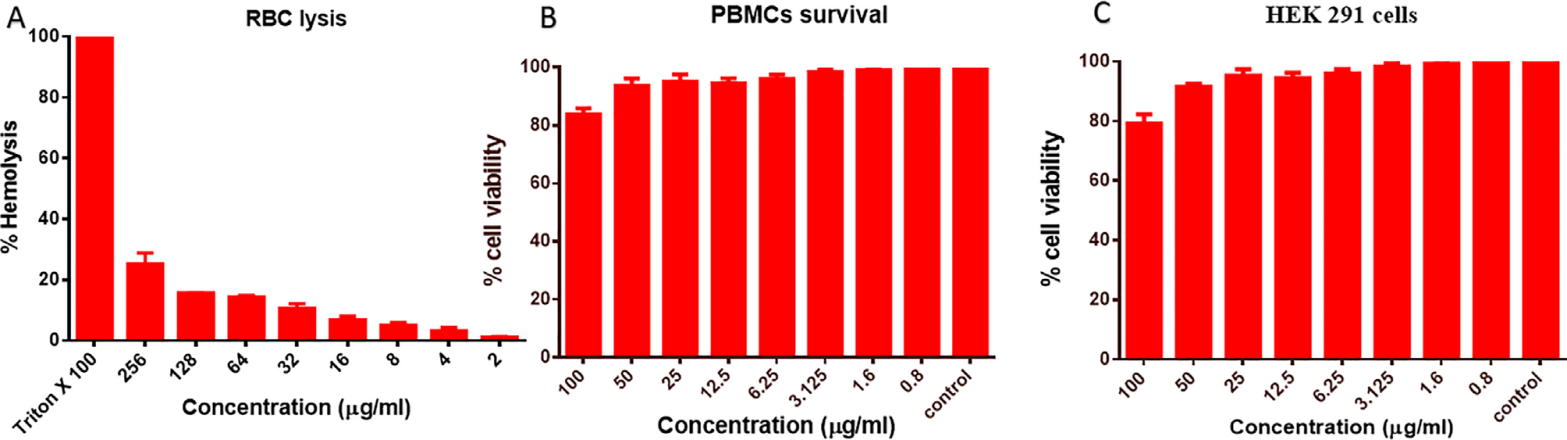
Cytotoxicity assays. **(A)** RBCs lysis assay when RBCs were co-cultured with different concentrations of B-AgNPs. Triton-X-100 (1%) and phosphate-buffered saline (PBS) were used as a positive and negative control. **(B)** Effect of AgNPs on the peripheral blood mononuclear cells (PBMCs). **(C)** HEK-291 cells after the exposure of varying concentration of B-AgNPs as detected by thiazolyl blue tetrazolium bromide (MTT) assay.

In order to study the biocompatibility, we carried out the toxicity assays on RBCs, PBMCs, and HEK-291 cell lines; the results obtained showed that even at a very high dose of the exposure to AgNPs, it showed the limited lysis of only 23.2% cells, as shown in **Figure 7A**; similarly, for PBMCs, it showed around 81% cell viability after an exposure to 100 µg/ml (**Figure 7B**), and 79% cell survival % (*p* ≤ 0.001) was observed, for HEK-291 cells (**Figure 7C**). The results obtained showed the biocompatibility of the synthesized nanoparticles in normal cells.

## Discussion

### Bacteria Isolation and Characterization

In this study, marine bacteria from 12 m deep seafloor of the Red Sea, near Jeddah coastal area were isolated. Most of the bacteria are halophilic and thermotolerant. In other studies, *Mesoflavibacter* sp. has been recovered isolated from the deep-sea hydrothermal vent (Stephen and Phillips, 2016). Among the 50 isolates strain, CEES51 was a maximum similarity with iodide-oxidizing bacteria and a maximum likelihood with *Flavobacterium* sp. and maximum relatedness with the iodide-oxidizing bacteria, *Mesoflavibacter zeaxanthinifaciens*. The most conserved sequences of a CEES51 strain have a pairwise sequence similarity of 99% with *Mesoflavibacter zeaxanthinifaciens* (EF 660753.1), and the phylogenetic tree of *Mesoflavibacter zeaxanthinifaciens* was constructed by the neighbor-joining method by MEGA 7.06 program (Nicolaus et al., 2010), as depicted in **Figure 1B**.

### Bacterial Exopolysaccharide Production

Hypersaline marine bacteria are a unique source of polysaccharides. These polysaccharides can be used in food stabilizers, pharmaceutical industries, and renewable energy. In this study, marine bacteria were isolated by EPS production from the 12-m deep surface of the ocean. Similarly, polysaccharide was also produced from marine bacteria, which are used in the manufacturing of potential therapeutic agents (Zhang et al., 2015). Marine bacteria mainly produce heteropolysaccharides that are made from three or four different backbone chains of monomers (Poli et al., 2010). Marine microbial EPS commonly constitutes the following monomer units: d-arabinose, d-ribose, and d-xylose as pentoses sugar; d-glucose, d-galactose, d-mannose, d-allose, l-rhamnose, and l-fucose as hexoses sugar, while uronic acid and d-glucosamine, d-galactosamine, d-glucuronic acids, and d-galacturonic acids as amino sugars. These sugars were found with inorganic group: phosphate, sulfate, and organic groups succinic, acetic, and pyruvic acids. EPS mainly produces by bacteria due to biotic and abiotic stress and provide the ability of adaptation to an extreme environmental condition like high salinity and temperature and low availability of a nutrient in surroundings (Kanmani and Lim, 2013). In advance, they provide the best adhesive nature of bacteria and support in the storage of carbon source and virulence (Raguenes et al., 1996). The elemental analysis of EPS revealed that the total biomass of around 35.66% were carbon, which is similar to the percentage of carbon (33.84%) in EPS produced by marine bacterial communities (Le Costaouëc et al., 2012). By carbon percentage data, the molecular mass of EPS is estimated to be in the range of 1 to 3 × 10^5^ Da (Thakore et al., 2014). The benefits of a marine bacterial source over the plant and animal source have made it smart to attain unique thermotolerant macromolecules for some industrial purposes in a sustainable manner.

### Synthesis and Characterization of Biogenic AgNPs

In this study, the nanoparticles are stabilized by the bottom-up approach and mediated by marine bacterial polysaccharide was stable and size within the range of about 35 nm. Nanoparticle synthesis was confirmed by UV-Vis spectrophotometer and other advanced characterization techniques (Kalishwaralal et al., 2010). The presented results were also similar to the previously published study of silver bio-mineralization from the EPS produced by marine bacterium *Alteromonas macleodii*. In another study, the aqueous extract from the *Marinobacter lipolyticus* has been utilized in silver nanoparticles preparation (Oves et al., 2016). The present study has a similar observation with nanoparticles prepared by Mehta et al. from the marine bacterial polysaccharide. Most of the previous studies on the synthesis of nanoparticles employing the bacterial polysaccharide indicated the polydisperse nature of the materials (Yang et al., 2009; Kumar and Mamidyala, 2011; Singh et al., 2014; Thomas et al., 2014); however, the present study represents monodisperse spherical-shaped nanoparticles. Here, XRD analysis was utilized to assure the crystalline nature of synthesized particles, and peek reflection represents the oval shape of silver by previous reports (Morones et al., 2005; Składanowski et al., 2016). The IR spectra revealing the involvement of the biological functional groups in nanoparticles synthesis is also in good agreement with previous reports by Kumar and Mamidyala (Kumar and Mamidyala, 2011).

### Antibacterial and Antibiofilm Assay

The biogenic synthesized silver nanomaterial was found to be more effective against *Bacillus subtilis* compared with *Staphylococcus aureus*. Antibacterial activities of biogenic silver nanoparticles performed on the solid agar medium were excellent due to the diffusion of nanoparticles in surrounding the well and prevented the growth of the bacterial cell. These findings are strong evidence with the study of green synthesized nanomaterial antimicrobial activity in the form of zone inhibition against *Pseudomonas aeruginosa,* *Salmonella* *paratyphi*, *Salmonella typhi*, *Staphylococcus aureus*, and *Vibrio cholerae* (Ashkarran et al., 2012; Martínez-Robles et al., 2016). Bactericidal property can be recognized by the strong interaction between the thiol group of membrane proteins and silver ions. The proteins become denatured and lose functioning, and the silver ions enter into cells and effect on DNA replication (Qayyum et al., 2017). Further checked MIC and MBC of synthesized nanomaterials, it was excellent against both bacteria 12.5 and 50 µg/ml individually. In other studies, MIC and MBC of nanomaterials have been reported against microorganism (Qayyum et al., 2017). Similar results have been reported by Składanowski (Składanowski et al., 2016). Furthermore, green synthesized nanomaterials were applied against biofilm and were found to have up to >80% reduction in the biofilm formed by *S. aureus*. These findings are in agreement with the previous studies reported by Ashkarran et al. (Ashkarran et al., 2012). In addition, our results were similar to another investigation of silver nanomaterials’ effect on *S. mutans* biofilm (Hodges et al., 1999). To confirm the deformation of biofilm and cell lysis by MDA formation and lipid peroxidation, *B. subtilis* and MRSA bacterial culture were treated with 100 µg/ml AgNPs in growing media; it produced MDA to 3.1 and 2.7 nm/cell dry wt., respectively. Earlier studies have confirmed that MDA is formed during lipid peroxidation of unsaturated fatty acid by free radicals, and MDA concentration is directly proportional to the amount of generated ROS that leads to cell deterioration. Here, MDA production results indicate that increasing concentration of AgNPs and incubation time enhance the free radical production in media. The MDA data correlate well with bacterial growth inhibition due to the generation of free radicals and damage of the membrane in the presence of AgNPs. Our result is similar to the previous study on the effect of nanoparticles on MDA production in treated *E. coli* (Hodges et al., 1999). Our study illustrates that novel bacterial EPS-based silver nanomaterials might provide great potential for the control of biofilm formation and bacterial infection and hence prevent infectious disease. Our future work will involve the employment of nanoparticles against infectious diseases in mice models and study the mechanism of their activities on different genes. However, more research and development is required to transfer the technology into pharmaceutical industries and microbe control strategies.

## Conclusion

In this study, EPS producing hypersaline marine bacteria were recovered from the 12-m deep Red Sea surface from Jeddah, KSA. In general, Red Sea beneficial microbes are still less explored; the recovered potential strain was purified and identified by the standard method of biochemical and molecular techniques. The present investigation was emphasized on the antibacterial properties of biogenic silver nanoparticles, which synthesized the EPS of *Mesoflavibacter zeaxanthinifaciens*. EPS application in capping or fabrication of silver nanoparticles were at room temperature without application of reducing chemical agent. This bacterial strain is capable of producing up to 32.5 g/L EPS after a 72-h incubation at optimum conditions. Synthesized nanoparticles were characterized using modern technology, and it was found to be a spherical shape and 35 nm in size. Furthermore, these nanoparticles were assessed as an antimicrobial agent against *B. subtilis* and MRSA. These AgNPs showed excellent antibiofilm activity against the tested strains. The antimicrobial action was confirmed by ROS generation during treatment. Therefore, the isolated marine bacteria can be used in EPS production and further for nanoparticle preparation, and these nanomaterials might be used for drug development and many other commercial purposes.

## Author Contributions

MO and MAR developed and designed the study. HAQ carried out the marine water sample for hypersaline bacteria isolation. AAPK, AH, and PM help in FTIR and XRD analysis. AH, MTR, MFA, and IIMI provided technical advice and culture media in the study. MO and MAR have also interpreted the data, drafted the manuscript. All author read and approved the manuscript.

## Conflict of Interest Statement

The authors declare that the research was conducted in the absence of any commercial or financial relationships that could be construed as a potential conflict of interest.
