# Supplementary material for: Antibacterial Silver Nanomaterial Synthesis From Mesoflavibacter zeaxanthinifaciens and Targeting Biofilm Formation
Source: Front Pharmacol. 2019 Aug 2;10:801. doi: 10.3389/fphar.2019.00801 (PMC6688106; doi:10.3389/fphar.2019.00801)
Supplement: Supplementary file 1 [file DataSheet_1.docx]

LOCUS MH707257 745 bp DNA linear BCT 08-AUG-2018

DEFINITION Mesoflavibacter zeaxanthinifaciens 16S ribosomal RNA gene, partial

sequence.

ACCESSION MH707257

VERSION MH707257.1 GI:1440885400

KEYWORDS .

SOURCE Mesoflavibacter zeaxanthinifaciens

ORGANISM Mesoflavibacter zeaxanthinifaciens

Bacteria; Bacteroidetes; Flavobacteriia; Flavobacteriales;

Flavobacteriaceae; Mesoflavibacter.

REFERENCE 1 (bases 1 to 745)

AUTHORS Oves,M.

TITLE Antibacterial silver nanomaterials synthesis from CEES51 strain and

targeting biofilm formation

JOURNAL Unpublished

REFERENCE 2 (bases 1 to 745)

AUTHORS Oves,M.

TITLE Direct Submission

JOURNAL Submitted (01-AUG-2018) CEES KAU, King Abdulaziz University,

Building Number 4, Center of Excellence in Environmental studies,

King Abdul Aziz University, Jeddah, Makkah 21589, Saudi Arabia

COMMENT ##Assembly-Data-START##

Sequencing Technology :: Sanger dideoxy sequencing

##Assembly-Data-END##

FEATURES Location/Qualifiers

source 1..745

/organism="Mesoflavibacter zeaxanthinifaciens"

/mol_type="genomic DNA"

/strain="CEES51"

/isolation_source="Red Sea water"

/db_xref="taxon:393060"

/collected_by="Mohammad Oves"

rRNA <1..>745

/product="16S ribosomal RNA"

ORIGIN

1 gatgaacgct agcggcaggc ttaacacatg caagtcgagg gggtaacaga gaaaagcttg

61 cttttttgct gacgaccggc gcacgggtgc gtaacgcgta tgcaatctac cttttgctga

121 gggatagccc agagaaattt ggattaatac ctcatagtat ggtgacttgg catcaagata

181 tcattaaagg ttacggcaaa agatgagcat gcgttctatt agctagttgg tgtggtaacg

241 gcataccaag gcaacgatag ataggggtcc tgagagggag atcccccaca ctggtactga

301 gacacggacc agactcctac gggaggcagc agtgaggaat attggacaat ggaggcaact

361 ctgatccagc catgccgcgt gcaggaagac tgccctatgg gttgtaaact gcttttatac

421 aggaagaaac acctctacgt gtagaggctt gacggtactg taagaataag gatcggctaa

481 ctccgtgcca gcagccgcgg taatacggag gatccaagcg ttatccggaa tcattgggtt

541 taaagggtcc gtaggtggat aattaagtca gaggtgaaat cctgcagctt aactgtagaa

601 ttgcctttga tactggttgt cttgagttat tatgaagtag ttagaatatg tagtgtagcg

661 gtgaaatgca tagatattac atagaatacc aattgcgaag gcagattact aataatatac

721 tgacactgat gaacgaaaag agcgg

//
