# Supplementary material for: Antibacterial Silver Nanomaterial Synthesis From Mesoflavibacter zeaxanthinifaciens and Targeting Biofilm Formation
Source: Front Pharmacol. 2019 Aug 2;10:801. doi: 10.3389/fphar.2019.00801 (PMC6688106; doi:10.3389/fphar.2019.00801)
Supplement: Supplementary file 2 [file DataSheet_2.docx]

**
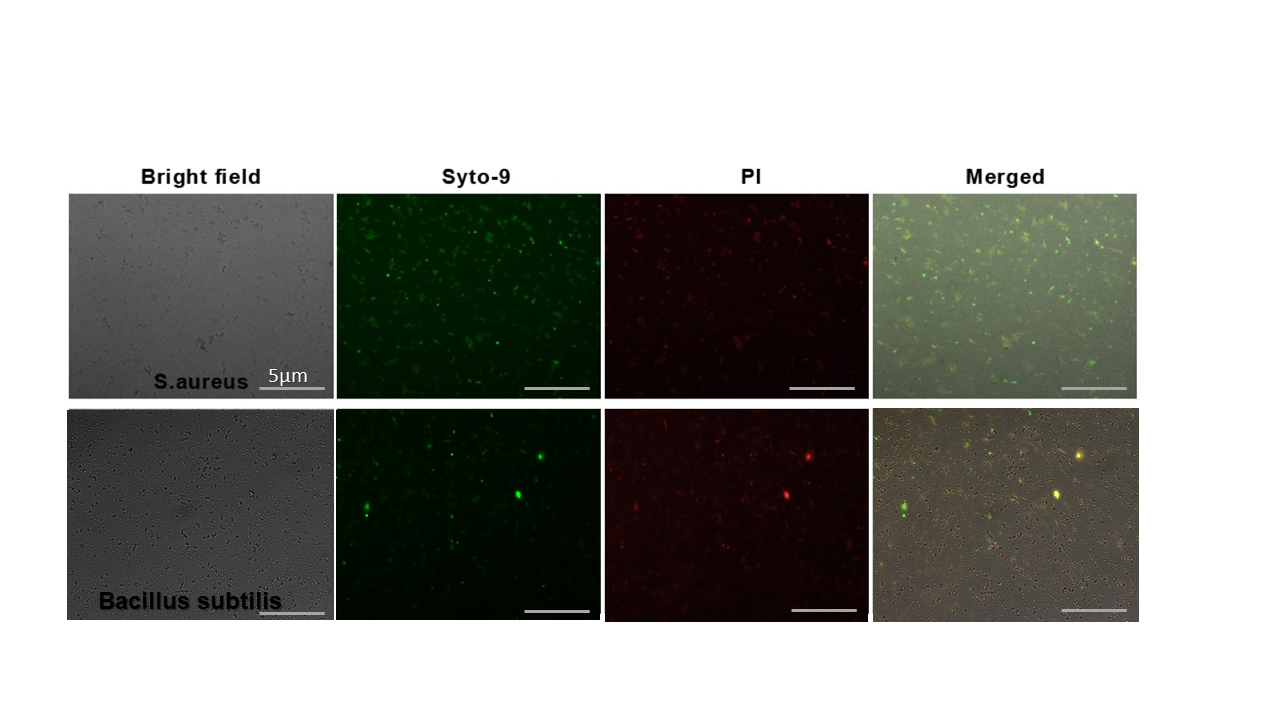
**

**Figure 1S.**The live-dead assay is employing Syto-9 and PI dye based Fluorescence microscopic images for MRSA and *B.subtilis* cells upon their treatment with AgNPs.
